# Deep Learning for Real-Time 3D Multi-Object Detection, Localisation, and Tracking: Application to Smart Mobility

**DOI:** 10.3390/s20020532

**Published:** 2020-01-18

**Authors:** Antoine Mauri, Redouane Khemmar, Benoit Decoux, Nicolas Ragot, Romain Rossi, Rim Trabelsi, Rémi Boutteau, Jean-Yves Ertaud, Xavier Savatier

**Affiliations:** Normandie University, UNIROUEN, ESIGELEC, IRSEEM, 76000 Rouen, France; antoine.mauri@esigelec.fr (A.M.); benoit.decoux@esigelec.fr (B.D.); nicolas.ragot@esigelec.fr (N.R.); romain.rossi@esigelec.fr (R.R.); rim.trabelsi@esigelec.fr (R.T.); Remi.Boutteau@esigelec.fr (R.B.); Jean-Yves.Ertaud@esigelec.fr (J.-Y.E.); xavier.savatier@esigelec.fr (X.S.)

**Keywords:** object detection, tracking, distance estimation, localisation, deep learning, smart mobility, 3D multi-object

## Abstract

In core computer vision tasks, we have witnessed significant advances in object detection, localisation and tracking. However, there are currently no methods to detect, localize and track objects in road environments, and taking into account real-time constraints. In this paper, our objective is to develop a deep learning multi object detection and tracking technique applied to road smart mobility. Firstly, we propose an effective detector-based on YOLOv3 which we adapt to our context. Subsequently, to localize successfully the detected objects, we put forward an adaptive method aiming to extract 3D information, i.e., depth maps. To do so, a comparative study is carried out taking into account two approaches: Monodepth2 for monocular vision and MADNEt for stereoscopic vision. These approaches are then evaluated over datasets containing depth information in order to discern the best solution that performs better in real-time conditions. Object tracking is necessary in order to mitigate the risks of collisions. Unlike traditional tracking approaches which require target initialization beforehand, our approach consists of using information from object detection and distance estimation to initialize targets and to track them later. Expressly, we propose here to improve SORT approach for 3D object tracking. We introduce an extended Kalman filter to better estimate the position of objects. Extensive experiments carried out on KITTI dataset prove that our proposal outperforms state-of-the-art approches.

## 1. Introduction

Advanced Driver Assistance Systems (ADAS) aims to blend algorithms and sensors to analyse the vehicle environment in order to provide to the driver valuable information so that he can be notified of potential hazards and for better assistance. For this, vision is the most important sense needed when driving; hence, computer vision is of great importance in such context so that we can process and understand automatically aforesaid visual data.

**Figure 1:**
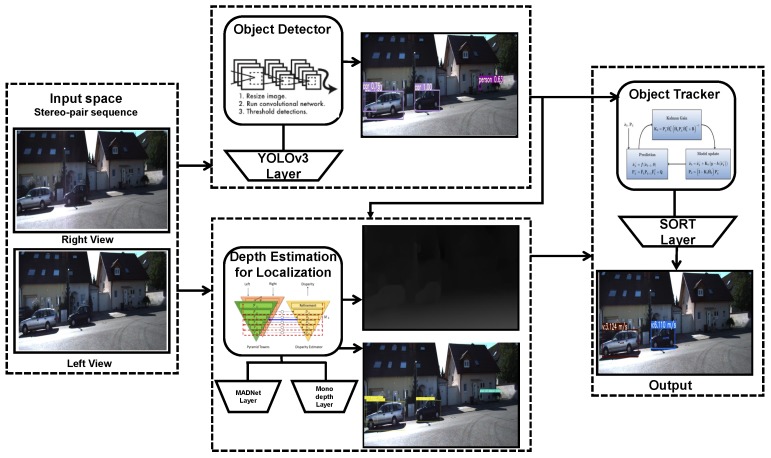
Overview of the proposed system composed of three main components: object detection, depth estimation and localisation and tracking.

In this context, the work presented in this paper deals with the task of multi-object detection, localisation and tracking for smart road mobility. Specifically, we propose a comprehensive system with various modules (cf. Figure 1) devoted to analyse the road environments.

Expressly, the main contribution of this paper is to introduce a new object detection, localisation and tracking technique dedicated to the smart mobility applications like traffic road environment. In smart mobility applications, the perception of the environment can significantly improve safety, especially in the detection of pedestrians, vehicles and other moving objects. In addition, logistical and technical problems continue to hamper industrial development in passenger and freight transportation. Recent endeavours [1,2] did show that technology can significantly improve the competitiveness of road transport by integrating innovative services but this area is still facing real issues and important challenges. Thus, our contribution is a unified framework that works on complex road environments to improve security, but also to make the traffic more fluent.

Since 2012, deep learning approaches have reported state-of-the-art results for most of the challenging tasks in computer vision area. We propose here to exploit the maturity of such methods in order to enhance the perception and the analysis of a niche area of application and road scene understanding. In this context, several works related to object detection, recognition and tracking have already been carried out in collaboration with SEGULA technologies, such as the tracking of a person by a drone [3], the detection and tracking of objects for the road smart mobility [4]. The goal is to propose new approaches of embedded vision dedicated to the detection of objects, their localisation as well as their tracking, which allow better performance in realistic and complex conditions.

The remainder of this paper is organized as follows. In Section 2, we review the state of the art of object detection and tracking based deep learning. The architecture of our proposed technique is introduced in Section 3. Our deep model of detection is then detailed in Section 4. Section 5 illustrates the object distance estimation approaches based on both monocular and stereoscopic images. The object localisation proposal is elaborated afterward in Section 6. The object tracking algorithm based on Extended Kalman Filter (EKF) [5] is introduced in Section 7. Experimental results for the above five proposed modules are reported along with the presentation of each approach respectively in Section 4, Section 5, Section 6 and Section 7. Finally, conclusions and future directions are drawn in Section 8.

## 2. Related Work

In what follows, we are going to review closely related work while highlighting the differences with our proposal in each of the following modules.

**Object detection.** is one of the main tasks of computer vision. Its challenges are mostly related to object localisation and object classification. Several methods based on Convolutional Neural Network (CNN) have been proposed over the past few years to overcome these challenging issues. CNN based methods have two main categories: (i) one-stage methods, which perform the object localization and object classification in a single network, and (ii) two-stage methods, which have two separated networks for each of these tasks. Among the one-stage state-of-the-art methods, we can cite Single-Shot Detector (SSD) [6] and You Only Look Once (YOLO) [7]. The output of both architecture is the bounding boxes each detected object, its class and its confidence score. As for the two-stage category, RCNN (Region-proposal CNN) [8] is reporting outstanding results over many benchmarks along with its improved versions [9,10], which are based on two independent neural networks, i.e., a region-proposal network and a classification network. They do report state-of-the-art results in terms of accuracy, especially for the localisation of objects. The performance of these approaches is evaluated using the mean Average Precision metric, a criterion which quantifies quality of detection (proportion of correct detection) as a function of the recall, which is the proportion of objects that are detected. The minor drawback of such networks is the use of two independent networks which increase the computationnal time, especially for embedded computation platforms. On the contrary, with the one-stage architectures, the classification is made on predefined size and number of bounding boxes at specific layers, then tune the localization of detected objects by regression. They are generally faster than two-stage ones, with similar performance [6]. Given that our application is based on spatio-temporal data, i.e., videos, inaccurate detection of too small objects and imprecise localisation are less important than real-time performance. Some trade-off like increasing number of bounding box and resolution of inputs also could be adopted to reduce it. Based on the above analysis, we choose to make use of the state-of-the-art one-stage object detection approach YOLOv3 [11].

**Distance estimation.** To estimate distance from vehicle to the scene’s objects, many sensors are available, e.g., laser-ultrasonic [12] and time of flight sensors [13]. These kinds of sensors are widely used in such contexts, yet the use of multiple types of sensors, distance sensors with cameras makes the system more complex and expensive. Unlike most of the closely related work and in order to relax the computational complexity, we investigate here in this work a monocular camera to infer the distance information of the scene used in the detection step. For learning purposes, if the datasets used for object detection had the distance from object as ground-truth information, information of distance could be learned by adding for example a regression output to the CNN. The literature still lacks such a dataset that contains the distance map for each instance. Another problem is the fully connected layer of the neural networks, i.e., the label class is discrete yet the label distance is continuous. To deal with this issue, there must be a layer of regression and a layer of classification at the end of the network. In addition, going for an unsupervised scheme, it might be an interesting solution to estimate depth for monocular images, like the one called a Monodepth [14] model, which is trained mainly on stereo images but infers disparity maps from monocular images.

**Object Tracking.** In the last few years, MOT (Multi-Object Tracking) based on deep-learning has reached state-of-the-art performance in terms of quality of tracking [15]. For example, an object detector like Faster-RCNN [10] associated with a linear Kalman filter allows a good compromise between processing time and quality of tracking, as shown by SORT (Simple Online and Realtime racking) [16].

In order to evaluate performance of tracking, we have to define what are the possible errors. A first type of error is a miss that is an object which exists in a sequence of images but which is not detected in one or more images. A second type is false positives, when a detected object is associated by the tracker to a ground-truth object and trajectory, but do not correspond to an existing object. A third type is mismatches, when detected objects correspond to existing ones but are not associated by the tracker to the correct ones. The sum of these three types of errors computed over the total number of objects present in all frames defines the Multi Object Tracking Accuracy (MOTA) [17], which is a commonly used criterion for tracking.

## 3. System Architecture: Materials and Methods

In this section, an overview of our designed system is presented along with the used hardware and software tools to build our three modules-based architecture.

### 3.1. Overview

In order to design an efficient object detection technique, achieving the right speed/accuracy trade-off for a given system is required. As hinted earlier, deep learning based approaches are reporting state-of-the-art results for most challenging contexts; however, the main constraints related to the use of Deep Learning algorithms is the computation cost required for training on large scale dataset. Although some deep architecture applied to different different domains can run on CPUs, for our task here, a fairly powerful Nvidia${}^{®}$ GPU with at least 4 GB of VRAM is crucial.

To perform the training of different deep learning architectures, the supercomputer MYRIA (https://www.criann.fr/renouvellement-2016/) is utilized, which is used by numerous research institutions and companies for applications ranging from fluid mechanics to Deep Learning calculations. MYRIA is composed of 366 Broadwell dual processor compute nodes (28 cores at 2.4 GHz, 128 GB DDR4 RAM) including 20 nodes dedicated to Deep Learning calculations, and each of them is equipped with either 4 GPU Kepler K80 (12 GB VRAM per GPU) or 2 GPU Kepler P100 (12 GB VRAM per GPU). For testing purposes, we make use of both servers equipped with 2 GPU GTX1080Ti each with 11 GB of VRAM and a computer with GPU GTX 1050 4 GB, 16 GB of RAM memory and CPU i5 8300 h.

### 3.2. Datasets

The second most important constraint in Deep Learning is the acquisition of large scale datasets needed for training and testing the performance of the proposed algorithms against the state-of-the-art ones.

Owing to the importance of autonomous driving, there are numerous datasets devoted to the road domain such as KITTI [18] which provides images from a stereoscopic camera, the depth of the scene measured by a Lidar Velodyne as well as annotations of the vehicles and pedestrians for the detected objects. Along with KITTI, many other datasets are available such as CityScapes [19], Pascal VOC [20], MS-COCO [21], ImageNet [22] and OpenImages [23].

### 3.3. Acquisition System

To carry out tests in real conditions, we make use of Intel RealsenseTM D435 cameras (Santa Clara, CA, USA). These sensors have the ability to provide what is called a depth map providing 3D information related to the distance of the surfaces of filmed objects from a specific viewpoint. These cameras have been used in [24] to make visual SLAM (Simultaneous Localisation and Mapping), or cartography and simultaneous localisation. This visual sensor has the ability to provide reliable results when used indoors but has not been tested in outdoor conditions.

### 3.4. Deep Learning Libraries

We employ several libraries available in the field of deep learning. Among the most important libraries, we can cite TensorFlow, which is the most famous open-source library. It was developed in Python and C ++ by Google. Similarly, Keras is an open-source deep learning library available also on Python. This library offers the possibility to create high-level neural networks. It is one of the easiest to understand and use, and it allows fast creation of network prototypes. However, it is less efficient than other recent libraries, for e.g., Pytorch, which is a high-level open-source library that has been developed to integrate seamlessly with other Python modules like Numpy, SciPy and Cython. This better integration makes this library simple to use while offering very good performance.

## 4. Object Detection Based on Deep Learning

### 4.1. Object Detection Based SSD

SSD [6] is based on a single network to perform the region proposal and classification, thus allowing to reduce the computational time. The feature extraction is performed by VGG-16 [25]. To allow a multi-scale detection, the size of the layers is gradually smaller, and each of them produces a separate prediction. Several implementations of SSD have been tested on Python. All tests were performed using a COCO-based pre-trained model consisting of 80 classes ranging from a person to a flower pot. The results of these different implementations in terms of computation time can be found in Table 1. An example of an image returned by the algorithm can also be found in Section 4.4. It is important to note that the detection performance is identical while the computation time and accessibility to code changes are affected in the different implementations.

### 4.2. Detectron Object Detection

**Figure 2:**
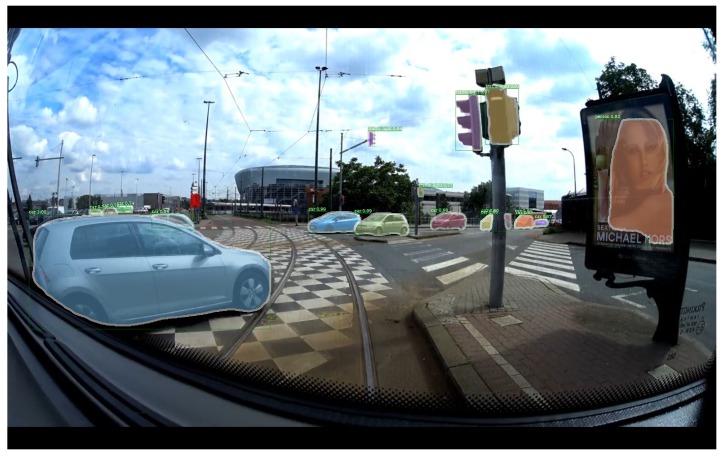
Mask-RCNN results (returns the mask of each detected object with their class and their confidence score).

Detectron is a C ++ and Python library specialized in object detection based on Deep Learning developed by Facebook and implemented in Caffe2. Among the present methods, we can find Faster RCNN [10] and Mask RCNN [8]. Both of these methods rely on two separate neural networks to perform the detection. The first one is the Region Proposal Network (RPN) [26] in charge of finding the region of interest where an object is located. The second network is for object classification in the bounding boxes returned by the RPN. Mask RCNN [8] is able to to extract a mask from the detected objects, which therefore represents a better localisation of the object in the image compared to the bounding boxes returned by most other object detection algorithms. However, this performance in terms of detection has heavy computing time and thus Mask RCNN [8] rotating at 3 frames per second. The detection results of RCNN Mask are illustrated in Figure 2.

### 4.3. YOLOv3 Object Detection

**Figure 3:**
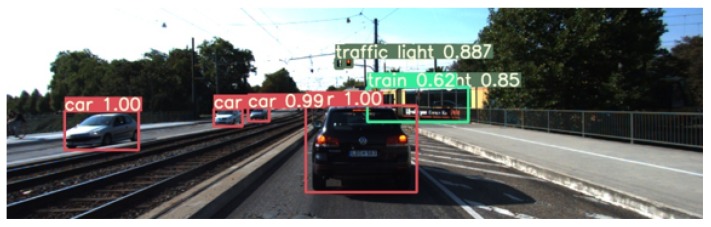
Result of YOLOv3 [2] (Returns the position, class and score of trust of each detected object).

Similar to SSD [6], YOLOv3 is a detector relying on a single network to perform the region proposal and the classification. The feature extraction is performed by DarkNet [27] with 53 layers. It has several implementations on different Deep Learning Python libraries. They have all been tested in order to compare their computation times. The obtained results are shown in Table 2 and Figure 3.

### 4.4. Object Detection Algorithm

#### 4.4.1. Selection Criteria

The selection criteria for the object detection method are based on their detection performance characterized by the mean average precision (mAP) metric as well as their response times. To be real-time, the computation time must be less than 100 ms or greater than 10 FPS.

#### 4.4.2. Performance of Processing Time

By relying on the tests of the different implementations of each method, one can already make a first selection. RCNN Mask is disqualified because it is well below 10 fps required for real-time use. Finally, we also deduce that the best implementation for YOLOv3 is the one on PyTorch and that the best implementation of SSD corresponds to the original implementation on Caffe.

#### 4.4.3. Performance of Detection

**Figure 4:**
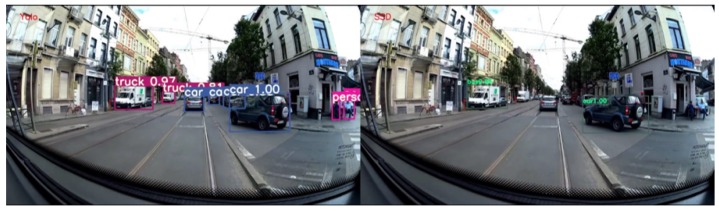
Performance comparison between YOLOv3 (**left**) and SSD (**right**) detectors.

Evaluating detection performance is a complex task that requires a lot of time. It is for this reason that we have relied on the results published in the article by YOLOv3 to choose the optimal algorithm. It indicates that YOLOv3 works better than SSD, which is confirmed by the inference tests we have done. Comparative results with state of the art can be found in Table 1. An example of detection of YOLOv3 with respect to the SSD is given in Figure 4.

In view of the above results, we therefore select YOLOv3 on PyTorch because it offers detection performance superior to that of SSD while offering an acceptable computational time. Finally, the advantage of YOLOv3 on PyTorch compared to SSD on Caffe is that the whole code is in Python, whereas Caffe is mainly in C ++, which makes YOLOv3 more simple to modify to match the constraints of the project.

## 5. Object Depth Estimation

We test several Deep Learning algorithms for distance estimation. For this purpose, we have focused on methods that do not require supervision during training because the ground truth for distance estimation is very complicated. Indeed, to obtain this ground truth from a lidar, it is necessary and yet it is bulky and extremely expensive. For our experiments, we make use of a KITTI dataset since it contains depth maps ground-truth.

### 5.1. Algorithm for Monocular Camera

**Figure 5:**
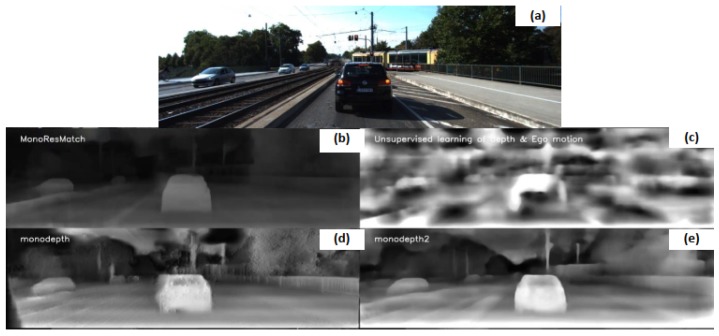
Results of disparity maps obtained using monocular approaches: (**a**) original image; (**b**) MonoResMatch; (**c**) SfmLearner; (**d**) Monodepth and (**e**) Monodepth2.

Among methods tested which are dedicated to monocular cameras, we can find approaches such as SfmLearner [28], MonoResMatch [29], Monodepth [14] and Monodepth2 [30]. Except [28], which is trained on monocular image sequences by learning the structure from motion elements using three frames snippet, all of these algorithms require a pair of images from a calibrated stereoscopic camera in order to be able to do the training. The training is generally performed by warping the right image features to the left; a reprojection error is then computed and used for the training loss. Afterward, inferences are performed on images from a monocular camera, which is a significant advantage because it reduces the cost due to equipment. Examples of disparity maps returned can be found in Figure 5.

### 5.2. Algorithm for Stereoscopic Cameras

**Figure 6:**
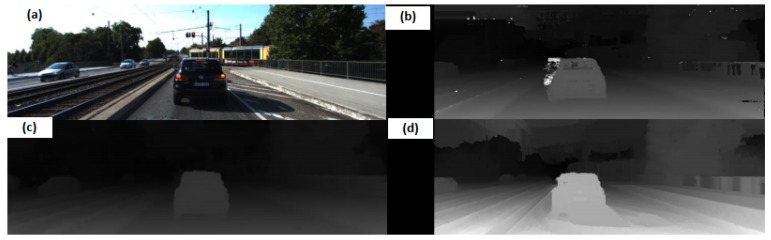
Results of disparity maps obtained using stereoscopic approaches: (**a**) original image; (**b**) stereo-baseline approach; (**c**) stereo-WLS filter and (**d**) MADNet.

Distance estimation by camera is often associated with the use of calibrated stereoscopic cameras. These classic methods are based on the association of pixels between the two cameras to obtain a map of disparity. The latter makes it possible to carry out the depth map. However, the association only works if the filmed scene is sufficiently textured, that is, it has enough features such as corners, edges, etc. This may be corrected using post-processing methods such as the WLS filter [31], but the quality of the results varies greatly. This is why Deep Learning algorithms have a real interest in getting the disparity map. We test here MadNet [32]; it has the particularity to be adaptive, i.e., it can perform the training at the same time as the inference and thus overcome a major defect of Deep Learning distance estimation algorithms: degraded performance on unknown environments. The method performs the adaptation by training to predict the disparity map between left and right images while inferring. It uses two pyramidal towers to extract the features from left and right frames, and each stage of the pyramids reduces the frame’s resolution. The loss is then computed with the reprojection errors between the warped right image and the left image for each stage of the pyramids. We have the choice between three modes during the inference: mode None to not do the adaptation, MAD mode to re-train on only part of the network or FULL mode to re-train the whole network. The MAD mode is a good compromise between times of calculation and distance estimation performance. Example of disparity maps returned by these methods can be found in Figure 6.

### 5.3. Choice of the Method

In order to select which method to use, we first determine which method performs best in each category: Monocular and Stereoscopic. The evaluation of the algorithms is problematic because each method returns the disparity map in a different format, so it is impossible to use it to find the depth map. To overcome this problem, each method has been tested on a sequence of the KITTI dataset because it has a ground truth of distance thanks to the lidar data. Owing to this, we can transform the disparity map returned by each method and transform it into a depth map using the median scaling between the inverse of the disparity predicted and the ground truth. The accuracy of the method is then estimated by computing the mean squared difference between the distance predicted by the algorithm and the ground truth. An example of distance estimation for objects can be found in Section 6.

#### 5.3.1. Monocular Approach

**Figure 7:**
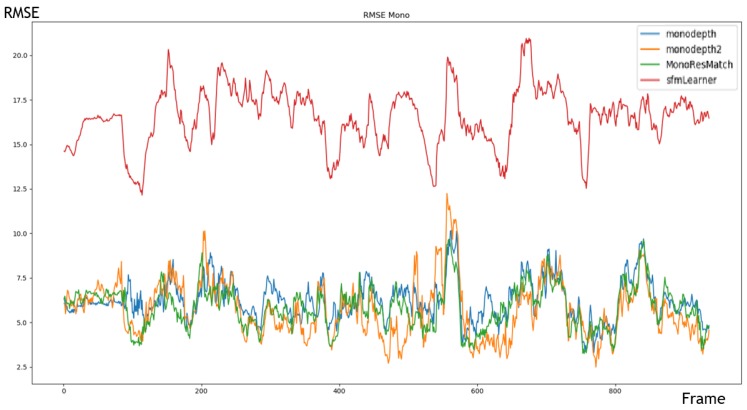
RMSE error over a sequence of arround 900 frames from the KITTI dataset. The blue, orange, green and red curves correspond respectively to the results of monodepth, monodepth2, MonoResMatch and sfmLearner approaches.

Considering the comparison results shown below, it appears that Monodepth2 [30] is the most suitable method if a monocular camera is used. This offers the best performance in terms of estimating distance and calculation time. However, it is important to note that Monodepth2 has been trained on the KITTI dataset and this one offers less good results on an unknown sequence. In both Figure 7 and Table 3, results and comparison with the state-of-the-art approaches in terms of RMSE (Root-Mean-Square Error) between the estimated distance (given by the algorithm) and the ground truth for each pixel of a given frame.

#### 5.3.2. Stereoscopic Approach

**Figure 8:**
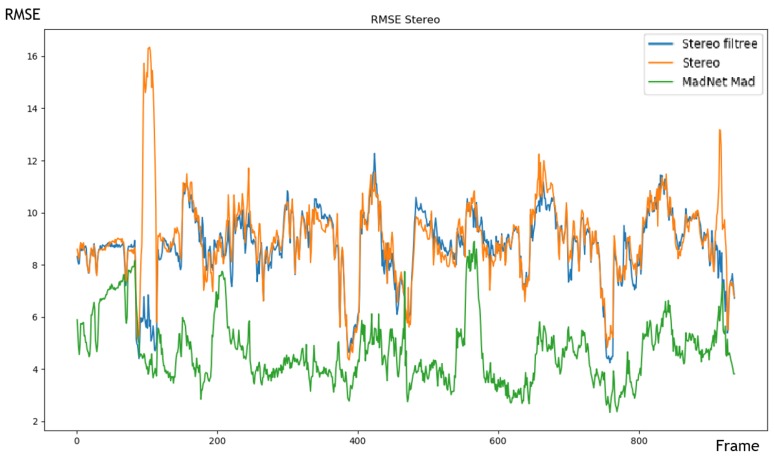
RMSE error over a sequence of around 900 frames from the KITTI dataset. The blue, orange and green curves correspond respectively to the results of Stereo-WLS Filter, Stereo-baseline and MADNet approaches.

Despite a computing time slightly short of real time (10 fps), the results in Figure 8 and in Table 4 show that MadNet offers performance in distance estimation superior to all other tested methods. It also has the advantage of being able to adapt to new environments with a stereo that allows more robust estimation than monocular methods. It can be used in indoor environments. Finally, the calculation time of MadNet can be reduced by slightly lowering the resolution of the input images at the cost of a slight decrease in performance of distance estimation but which remains marginal. We chose the MadNet method since it proves higher robustness and can adapt to new environments and can be used in real time if we decrease the resolution of the images.

## 6. Object Localisation

Thanks to the information provided by object detection (bounding boxes of objects) and depth estimation (depth map of the scene), it is now possible to locate each object on a 3D plane with respect to the camera’s coordinate system. Our problem boils down to projecting the coordinates in pixels of an object in the coordinate system of the camera.

**Figure 9:**
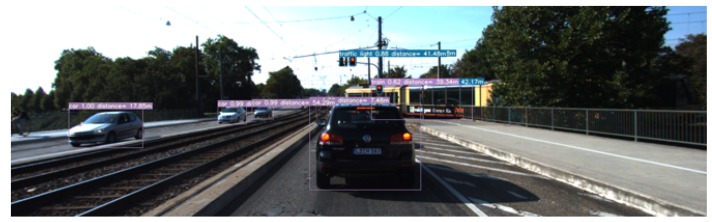
Object detection and localisation results over a sample from the KITTI dataset.

With $\left(o_{x},o_{y}\right)$, the coordinates of the optical center of the camera $\left(s_{x},s_{y}\right)$, the size of a pixel and *f* the focal length of the camera, the *Z* coordinates can be determined using the depth map information with the Pythagorean 3D theorem. Thus, we end up with a system of three equations with three unknowns, which allows us to find the coordinates of an object in the camera. In order to perform the localisation, we combine object detection and depth estimation in a single framework in order to check whether the real-time application is feasible. The program performs object detection and distance estimation; in parallel, it allows computational time reduction. The results can be found in Figure 9.

## 7. Object Tracking

### 7.1. 2D Tracking

As our application needs real-time processing and good performance, we have chosen the Simple Online Real-Time Tracking (SORT) algorithm [16], which represents a good compromise between those two aspects and is conceptually simple. This algorithm uses the bounding boxes provided by a detection algorithm to initialize and track targets based on a Kalman filter [33]. The Kalman filter has state vector *X* and measure vector *z*, defined by:

(1) $$\begin{array}{c} X=\left(x\;\;y\;\;r\;\;a\;\;\dot{x}\;\;\dot{y}\;\;\dot{a}\right){}^{t},\;\;z=\left(x\;\;y\;\;r\;\;a\right){}^{t}, \end{array}$$

where (*x*, *y*) are the coordinates of the bounding box, *r* its width over height ratio, and *a* its area. Quantities with a dot over them are for derivatives with respect to time. The prediction model is based on constant velocity.

The association between the targets and the detections is done by calculating the IOU (intersection over union) between a detection and the position predicted by the Kalman filter. If the detection gets an IOU greater than a predefined threshold and has the highest IOU among the other detections, then it is associated with the target, and the state vector of the Kalman filter corresponding to the target is updated. The performance of this algorithm depends directly on the quality of object detection and the computation time is less than 1 ms/frame. Although this method yields good results, given that it predicts the target’s next position in 2D, it lacks 3D information to perform well when the target is occluded.

### 7.2. 3D Tracking

In view of our goal to track objects in 3D, many changes have been made. Thus, the Kalman has been replaced by an extended Kalman filter. The main parameters can be found below:

(2) $$\begin{array}{c} X_{s}=\left(X\;\;Y\;\;Z\;a\;\;r\;\;\dot{X}\;\;\dot{Y}\;\;\dot{Z}\;\;\dot{a}\right){}^{t},\;\;z=\left(x\;\;y\;\;d\;\;r\;\;a\right){}^{t}, \end{array}$$

with (*X*, *Y*, *Z*) corresponding to the coordinates in 3D and d is the depth estimated by the detection. In order to make predictions, we use the constant velocity model. The measurement vector *z*, composed of the coordinates measured with object detection and distance estimation, allows us to update the filter status vector. We also tested the constant acceleration model, but predictions tend to diverge making tracking impossible. The model of constant acceleration could render better results than the constant velocity if the tracked objects change abruptly at regular intervals, which is not the case here because the extended Kalman filter has a sufficient sampling rate (in this work, it is equal to the frame rate). By using 3D coordinates instead of 2D, we further improved the prediction of the tracking of our method and made it more robust to a realistic environment.

### 7.3. Adjusting the Parameters of the Extended Kalman Filter

The control noise matrix adjusts the behavior of the filter. This way, the confidence rate between the measurements and the prediction model can be adjusted. To filter out a large measurement noise, it is better to reduce the confidence level of the measurements, but the filter will have more difficulties in the event of a change of trajectory. It is therefore a question of finding a balance between noise filtering and measurement monitoring. We used trial/error to find these parameters.

### 7.4. Object Tracking Results

**Figure 10:**
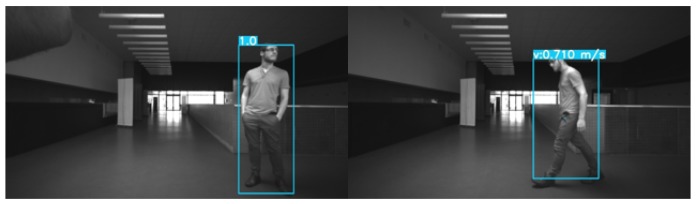
Result of the tracking approach over an indoor scene using the stereo images provided by the Intel RealSense D435 sensor. The left frame is acquired at instant t=1 and the right one at the instant t+3. At t=1, we assign an ID to each detected object. Then, at t+3, the tracklet is validated and we display the estimated speed.

We test the tracking algorithm in both indoor and outdoor environments. Initially, tests were performed indoors, the advantage being that it is easier to determine whether the predicted values diverge or not. The results can be found in Figure 10.

**Figure 11:**
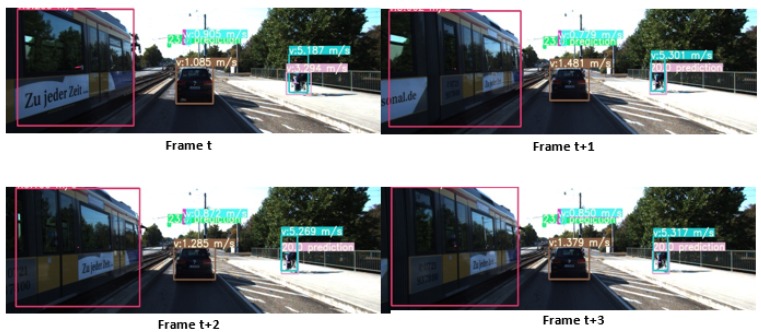
Tracking results in the road environment.

**Figure 12:**
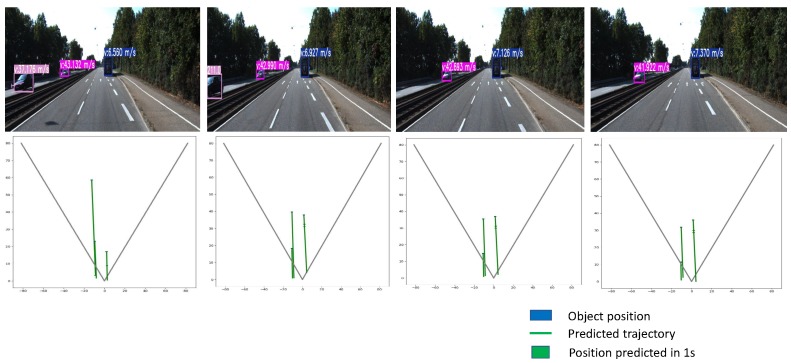
Tracking results over a sequence from the KITTI dataset. On top, four RGB frames coming from a road sequence acquired at different times with the corresponding tacking boxes of the moving objects and their speed values. On bottom, the maps shown are proposed by this work in order to generate a synthetic tool that allows simple yet comprehensive results overview of motions and distances of the tracked objects.

We then carried out tests in the road domain using the KITTI dataset (see Figure 11). Here, we can see that the bike on the right is no longer detected from the frame (t+1). The prediction of the Kalman filter is then used to estimate the position of this object. If this one is not associated with a detection before three frames, the algorithm deletes this target. Our method also allows for tracking a target even if it is not associated with a detect blob for up to three frames before it will be deleted. We can see in Figure 11 and Figure 12 quantitative and qualitative results of our method over two different sequences of KITTI datasets.

## 8. Conclusions and Future Directions

In this paper, we have presented an end-to-end deep learning based system for multi-object detection, depth estimation, localisation, and tracking for realistic road environments. For the object detection module, an effective detector based on YOLOv3 was proposed. Jointly, depth maps estimation has been introduced in order to render the localisation information of the detected objects. For this purpose, we make use of two different kinds of approaches, i.e., Monodepth2 and MADNet to design the second module of object localisation. Finaly, a new object tracking method is introduced based on an improved version of the SORT approach. Extended Kalman Filter is presented to improve the estimation of object’s positions. For each step, we set up various experiments over the large-scale KITTI dataset while comparing our proposal with the state-of-the-art approaches. Overall, the reported qualitative and the quantitative results prove that our deep learning proposal is robust enough against challenges of road scenes. Ultimately, we aim to use our solution in both indoor (such as smart wheelchair for disabled people [24]) and outdoor (soft mobility for car and tramway) environments. The road and tramway domains are quite similar to outdoor environments, which allows the algorithms trained on the road domain to offer good performances when inferring on the tramway domain. However, due to the lack of a publicly available dataset for railway environments with 2D and 3D informations (barring the recently published RailSem19 dataset [34]), we are in need of acquiring our own dataset for this task. For this, we propose to extend the current version of our system by including a new stereoscopic sensor so that we can collect our own dataset under outdoor conditions with large and adjustable baselines.

## Figures and Tables

**Table 1:** Performance evaluation of our SSD-based detector over KITTI dataset against state-of-the-art approaches.

Approach	mAP	FPS	Image Resolution
Faster R-CNN	73.2	7	1000 × 600
Fast YOLO	52.7	155	448 × 448
YOLO (VGG16)	66.4	21	448 × 448
SSD300	74.3	46	300 × 300
SSD512 (ours)	76.8	19	512 × 512

**Table 2:** Performance evaluation of our YOLOv3-based detector over a KITTI dataset against state-of-the-art approaches.

Approach	mAP-50	Run-Time (ms)
SSD321	45.4	61
SSD513	50.4	125
R-FCN	51.9	85
FPN FRCN	59.1	172
YOLOv3-320	51.5	22
YOLOv3-416	55.3	29
YOLOv3-608 (ours)	57.9	51

**Table 3:** Experimental results of monocular approaches.

Approach	RMSE	FPS
sfmLearner	16.530	20
Monodepth	6.225	5
MonoResMatch	5.831	1
Monodepth2	5.709	20

**Table 4:** Experimental results of stereoscopic approaches compared to our proposal based MADNet.

Approach	RMSE	FPS
Stereo-baseline	9.002	15
Stereo-WLS Filter	8.690	7
MADNet (ours)	4.648	7
